# Successful management of cryptococcosis of the bilateral adrenal glands and liver by unilateral adrenalectomy with antifungal agents: a case report

**DOI:** 10.1186/1471-2334-11-340

**Published:** 2011-12-14

**Authors:** Yayoi Matsuda, Hisaya Kawate, Yuka Okishige, Ichiro Abe, Masahiro Adachi, Keizo Ohnaka, Naoichi Satoh, Junichi Inokuchi, Katsunori Tatsugami, Seiji Naito, Masatoshi Nomura, Ryoichi Takayanagi

**Affiliations:** 1 Department of Medicine and Bioregulatory Science, Graduate School of Medical Sciences, Kyushu University, Fukuoka 812-8582, Japan; 2 Department of Geriatric Medicine, Graduate School of Medical Sciences, Kyushu University, Fukuoka 812-8582, Japan; 3 Department of Endocrinology and Diabetes, Iizuka Hospital, Iizuka 820-8505, Japan; 4 Department of Urology, Graduate School of Medical Sciences, Kyushu University, Fukuoka 812-8582, Japan

## Abstract

**Background:**

*Cryptococcus *species usually affect the central nervous system and lungs in immunocompromised hosts. Although the adrenal glands can be involved in disseminated cryptococcosis, primary adrenal insufficiency caused by the fungal infection is uncommon.

**Case presentation:**

We present a case of primary adrenal insufficiency with bilateral adrenal masses and liver invasion in a 43-year-old man with mild type 2 diabetes mellitus. Cryptococcosis was diagnosed by fine-needle aspiration biopsy of the liver mass. The serum cryptococcal antigen titer was elevated to 1:256. After 6 months of antifungal therapy with fluconazole and amphotericin B, the size of the liver mass was decreased, but no significant changes were observed in the bilateral adrenal masses and the serum cryptococcal antigen titer remained elevated at 1:128. To control the cryptococcosis, a laparoscopic left adrenalectomy was performed, followed by antifungal therapy. After the unilateral adrenalectomy, the size of the remaining right adrenal mass was reduced and the serum cryptococcal antigen titer declined to 1:4.

**Conclusions:**

This is the first report describing adrenal cryptococcosis with adrenal insufficiency and liver invasion without central nervous system involvement. Adrenal cryptococcosis should be considered in the differential diagnosis for patients with bilateral adrenal masses with primary adrenal deficiency. Unilateral adrenalectomy was quite effective in controlling the cryptococcosis in this case. Even in patients with bilateral adrenal cryptococcosis, unilateral adrenalectomy should be an option for treatment of disseminated cryptococcosis.

## Background

*Cryptococcus neoformans *is an encapsulated yeast-like fungus found in soil and in pigeon or other avian feces. Cryptococcus is believed to be acquired by inhalation of fungal spores into the respiratory tract [1,2]. Since cell-mediated immune responses contribute to protection against cryptococcus, patients with impaired cell-mediated immunity, such as advanced AIDS or lymphoid and hematopoietic malignancies, and transplantation recipients are particularly prone to development of cryptococcosis [2-4]. Another species, *Cryptococcus gattii*, that is relevant to a recent outbreak in western North America is known to cause cryptococcosis in immunocompetent individuals [1,2,5]. Pulmonary cryptococcosis and cryptococcal meningitis are common manifestations in immunocompromised hosts. Disseminated cryptococcosis in immunocompromised hosts less frequently affects the skin, eyes, lymph nodes, bone marrow, liver, spleen, adrenal glands, kidneys, prostate, kidneys, thyroid, intestine, pancreas and ovaries [4,5]. Cryptoccocal infection of adrenal glands and liver is usually associated with involvement of the central nervous system and the lungs [2,6]. Here, we report a case of mild diabetic 43-year male with cryptococcosis of the bilateral adrenal glands and liver with adrenal insufficiency without involvement of the lungs and central nervous system. Since long-term therapy with antifungal agents was not effective against his cryptococcosis, a unilateral adrenalectomy was performed. The sizes of both the adrenal and liver lesions were decreased after the surgery followed by antifungal therapy and the serum cryptococcus antigen titer of the patient was markedly reduced from 1:256 to 1:4.

## Case Presentation

A 43-year-old man with mild type 2 diabetes mellitus visited the hospital where he received his medications for diabetes mellitus, owing to persistent dizziness, anorexia and general fatigue for 1 month before the consultation. He showed significant weight loss of 15 kg during that 1 month. Approximately 2 weeks before the emergence of his clinical symptoms, he walked around a cave where he encountered a lot of bird droppings and feathers. Since abdominal CT revealed bilateral adrenal masses, he was introduced to a larger hospital for advanced examination. On admission, physical examination revealed a height of 167 cm and a weight of 62 kg. The remainder of the examination findings were normal without signs of meningitis. Laboratory data included white blood cell count of 7,390/μL with 61.7% neutrophils, 23.9% lymphocytes, 2.4% eosinophils, 0.5% basophils and 9.4% monocytes, hemoglobin of 12.6 g/dL and platelet count of 313,000/μL. Although the serum sodium level (133 mEq/L) was slightly decreased, the serum levels of potassium, chloride, creatinine and fasting glucose were normal. The HbA1c was 6.2%. The level of γ-gamma glutamyl transpeptidase was elevated to 116 U/L without increased levels of aspartate aminotransferase, alanine aminotransferase and alkaline phosphatase. The serum C-reactive protein level was elevated (5.04 mg/dL). On endocrinological examination, low basal serum cortisol (2.3 μg/dL; normal: 4.0-18.3) with high serum adrenocorticotropic hormone (ACTH) (843 pg/mL; normal: 7.2-63.3) was observed, indicating primary adrenal insufficiency. In addition to the low blood levels of aldosterone (28.5 pg/mL; normal: 38.9-307) and dehydroepiandrosterone sulfate (55 μg/dL; normal: 70-495) synthesized and released by the adrenal cortex, the 24-hour urine adrenaline (1.6 μg/day: normal: 3.4-26.9) and epinephrine (< 0.01 mg/day; normal: 0.04-0.19) levels were markedly reduced, indicating that the adrenal medulla was also devastated. Rapid ACTH (Cortrosyn) stimulation revealed the absence of a serum cortisol response (baselines of 1.1 μg/dL to 1.1 μg/dL and 1.1 μg/dL at 30 and 60 minutes after the ACTH challenge, respectively). Primary adrenal insufficiency was diagnosed and a regimen of oral hydrocortisone (20 mg/day) was prescribed.

**Figure 1:**
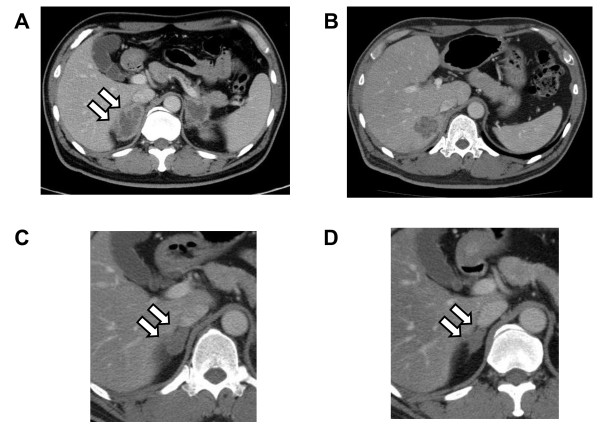
**Computed tomography of the abdomen**. (A and B) The enlarged bilateral adrenal glands (A) and liver mass (B) before the unilateral adrenalectomy. (C and D) Decreased size of the right adrenal mass at 3 (C) and 12 (D) months after the left adrenalectomy. The arrows show the right adrenal mass.

Contrast-enhanced abdominal CT showed bilateral adrenal masses (right: 5.2 × 2.7 cm; left: 3.7 × 3.6 cm) (Figure 1A). The differential diagnosis of the adrenal masses included metastatic carcinoma, tuberculosis, fungal infections, bilateral adrenal hyperplasia and sarcoidosis. An intensive whole-body examination failed to detect a primary lesion for malignancy. The QuantiFERON-TB test and serum human immunodeficiency virus (HIV) antibody enzyme immunoassay were negative. Re-examination of the abdominal CT at 1 month after admission revealed a liver abscess that was thought to be an invasion from the right adrenal mass (Figure 1B). Whole-body ^18 ^F-FDG PET showed intense uptake by the bilateral adrenal glands and liver.

To make a diagnosis, a fine-needle aspiration biopsy of the liver mass was performed. The pathological diagnosis of the liver abscess showed multiple foci of necrotic and degenerative cells with infiltration of neutrophils. No malignant cells or epithelioid granulomas were observed in this specimen. Alcian blue staining demonstrated the presence of 5-μm spherical yeast-like organisms, such as *Cryptococcus *spp., interspersed within the foci. Since the serum cryptococcal antigen titer was 1:256, cryptococcosis of the bilateral adrenal glands and liver was diagnosed. A lumbar puncture revealed clear cerebrospinal fluid with a white blood cell count of 1/μL, normal levels of protein and glucose and negative cryptococcal antigen titer.

After 4 months of fluconazole treatment at a daily dose of 400 mg, the size of the liver abscess was reduced, but no significant changes were observed in the bilateral adrenal masses and the serum cryptococcal antigen titer was still elevated at 1:128. Additional treatment with liposomal amphotericin B at 150 mg daily for 6 weeks (a cumulative dose of 6.3 g) was not effective.

**Figure 2:**
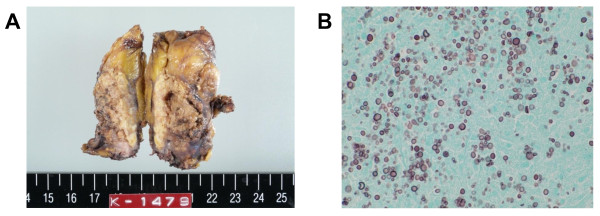
**Resected left adrenal mass**. (A) Gross appearance of the surgically resected left adrenal mass. (B) Histopathological analysis of the adrenal mass. Massively necrotizing granulomas and fibrous tissue with many cryptococcal cells were observed (Grocott stain, ×40).

Since it was conceivable that the bilateral adrenal glands were the apparent foci of the persistent fungemia, the patient was referred to our hospital and a laparoscopic left adrenalectomy was performed to control the cryptococcosis. Resection of the right adrenal mass was not executed as the first operation to avoid injury to the adjacent liver. The size of the resected mass was 5 × 4 cm (Figure 2A). Histological analysis revealed that the adrenal tissue was widely replaced by massively necrotizing granulomas and fibrous tissue. Many fungi structures, similar to *Cryptococcus *spp. cells were detected by PAS and Grocott staining (Figure 2B).

Fluconazole therapy was continued after the adrenalectomy. At 3 months after the adrenalectomy, a significant size reduction of the right adrenal mass was observed by abdominal CT (Figure 1C). The serum cryptococcal antigen titer also decreased to 1:16 at 2 months after the adrenalectomy, and 1:4 at 5 months after the operation. There were no signs of relapse in imaging analyses and the serum cryptococcal antigen titer remained at 1:4 for the subsequent 9 months (Figure 1D).

## Discussion

Cryptococcosis caused by *Cryptococcus neoformans *commonly affects the lungs and central nervous system, particularly in patients with HIV infection [4,5]. Although less frequent, cryptococcal infection also occurs in HIV-negative patients, including those with liver cirrhosis, systemic lupus erythematosus, malignancies and diabetes mellitus, and organ transplant recipients [7,8]. Even in mild diabetic patients like our patient, disseminated cryptococcosis has been reported [9,10]. Another species, *Cryptococcus gattii*, has recently been responsible for an outbreak of cryptococcosis in immunocompetent hosts in western North America [5,11,12]. Cryptococcosis due to *Cryptococcus gattii *is geographically restricted in Australia, Papua New Guinea, Canada and the Pacific Northwest of USA. In the present study, differentiation between *C. neoformance *and *C. gattii *has not been performed because *C. gattii *is an extremely rare in Japan. To date, only one case without a recent history of travel to disease-endemic area has been reported [13].

Although the adrenal glands are able to be involved in disseminated cryptococcosis, adrenal insufficiency caused by this fungus is uncommon [14]. In previous reports, most cases of adrenal insufficiency caused by cryptococcosis were accompanied by meningoencephalitis [9,15-20]. There are only two previous reports describing patients with isolated adrenal cryptococcosis without meningoencephalitis [9,20]. To the best of our knowledge, this is the first report of cryptococcosis of the bilateral adrenal glands and liver without meningoencephalitis. Since the liver mass was only observed adjacent to the right adrenal mass and its emergence lagged behind those of the adrenal tumors, we speculated that the cryptococcal cells in the right adrenal gland had directly invaded the liver and formed the lesion.

Several authors have reported that adrenal cryptococcosis presenting with adrenal insufficiency is refractory to antifungal chemotherapy [9,18]. In such cases, bilateral adrenalectomy was effective for controlling cryptococcal meningoencephalitis. In our case, antifungal therapy was effective in reducing the liver mass, but the bilateral adrenal masses did not change and the liver mass still existed. Since the adrenal masses were thought to be the foci of the persistent fungemia, a left adrenalectomy was performed. We chose a unilateral adrenalectomy because adhesion of the right adrenal mass to the liver was anticipated by imaging analyses and a right adrenalectomy may have caused massive bleeding.

The left adrenalectomy led to remarkable decreases in the serum cryptococcal antigen titers and a size reduction of the remaining right adrenal mass. We are continuing to carefully follow up the patient. If the masses in the adrenal gland or liver start to become enlarged or the cryptococcus antigen titer elevates, a right adrenalectomy and partial hepatectomy will be taken into consideration.

## Conclusions

This is the first report describing adrenal cryptococcosis with adrenal insufficiency and liver invasion without central nervous system involvement. If a patient has bilateral adrenal masses with adrenal insufficiency, adrenal cryptococcosis should be considered in the differential diagnosis. In this case, a unilateral adrenalectomy significantly decreased the size of the remaining adrenal mass and the cryptococcal antigen titer. When the effects of antifungal therapies are insufficient in patients with cryptococcosis of the bilateral adrenal glands, a unilateral adrenalectomy, as well as a bilateral adrenalectomy, should be an option for the treatment of disseminated cryptococcosis.

## Consent

Written informed consent was obtained from the patient for publication of this case report and any accompanying images. A copy of the written consent is available for review by the Editor-in-Chief of this journal.

## Competing interests

The authors declare that they have no competing interests.

## Authors' contributions

YM, HK, YO, IA, MA, KO, NS, MN and RT collected the patient data and participated in the treatment. YM and HK wrote the manuscript. MN and RT revised and edited the manuscript. JI, KT and SN performed the surgical treatment. All authors read and approved the final manuscript.

## Pre-publication history

The pre-publication history for this paper can be accessed here:

http://www.biomedcentral.com/1471-2334/11/340/prepub
